# Impact of menopause on responses to hypoxia and incidence of acute mountain sickness

**DOI:** 10.1007/s00421-025-05790-6

**Published:** 2025-04-29

**Authors:** Tom Citherlet, Antoine Raberin, Giorgio Manferdelli, Grégoire P. Millet

**Affiliations:** University of Lausanne, Institute of Sport Sciences, Synathlon, 1015 Lausanne, Switzerland

**Keywords:** Chemosensitivity, Aging, Menopausal, Pulse saturation, Ventilation, Women

## Abstract

**Purpose:**

Menopause results in decreased ovarian hormones, potentially impacting physiological responses to hypoxia and its tolerance. This study explored menopause’s influence on physiological responses during rest and exercise in normobaric hypoxia and its role in predicting acute mountain sickness (AMS).

**Methods:**

Thirteen eumenorrheic women in their mid-luteal phase (EW, age = 32 ± 8 year) and fifteen postmenopausal women (PW, age = 63 ± 2 year) were examined on two occasions. Their ovarian hormonal levels were measured. In the first visit, hypoxic ventilatory response (HVR), physiological responses (ventilation, pulse oximetry, and heart rate) at rest and exercise in normobaric hypoxia (F_i_O_2_ = 0.14) and anxiety levels were tested. On the second visit, cortisol awakening response and oxidative stress markers were measured at low altitude, with cortisol awakening response repeated during an overnight stay at high altitude (3375 m) along with evaluation for AMS using the Lake Louise Score, peripheral oxygen saturation and anxiety levels.

**Results:**

PW exhibited lower estradiol (16.9 ± 16.7 vs 4.6 ± 2.3 pg/ml, p < 0.01) and progesterone (13.39 ± 7.61 vs 0.06 ± 0.07 ng/ml, p < 0.001) levels than EW. Despite higher ventilation at rest in EW compared to PW in normoxia (10.0 ± 1.5 vs 8.5 ± 0.9 L/min; p < 0.01) and hypoxia (9.4 ± 1.3 vs 8.2 ± 1.3 L/min) , HVR (– 0.34 ± 0.13 vs – 0.27 ± 0.15 L/min/%) was similar between groups (p = 0.26). AMS incidence did not differ between EW (31%) and PW (40%).

**Conclusion:**

In conclusion, EW had higher ventilation at rest in normoxia and normobaric hypoxia compared to PW, but similar responses and AMS incidence at high altitude. Age has minimal impact on HVR in women.

## Introduction

Tolerance to hypoxia (i.e., the ability to adapt to lower oxygen availability), is critical for high altitude acclimatization and is primarily driven by cardiovascular and respiratory adaptations (Mallet et al. 2023). Among the factors influencing this tolerance, sex hormones may play a notable role, particularly by modulating the hypoxic ventilatory response (HVR). There has been a growing research interest in this topic (Raberin et al. 2023). Hormonal fluctuations associated with menstrual cycle (Schoene et al. 1981; White et al. 1983), pregnancy (Moore and McCullough 1985; Hannhart et al. 1989), estradiol treatment in postmenopausal women (PW; Cistulli et al. 1994), or ovariectomy (Tatsumi et al. 1997) have substantial effects on HVR (Raberin et al. 2023). Progesterone stimulates HVR by increasing carotid body sensitivity to hypoxia, and, in combination with estrogen, by possibly adjusting the central processing of signals originating from the carotid body (Hannhart et al. 1989). However, a recent study did not report any differences in HVR in eumenorrheic women between the different phases of the menstrual cycle (Citherlet et al. 2024).

Menopause, characterized by the cessation of menstruation, corresponds to a significant reduction of estrogen and progesterone levels and offers a natural model to assess the effects of sex hormones and, indissociably, of age on the physiological responses to hypoxia in women (Elliott-Sale et al. 2021). One may thus expect a change in HVR with hormonal variations linked to menopause . However, to date, most of the studies do not support the hypothesis of a decrease in HVR with menopause (Pokorski and Marczak 2003; Pokorski et al. 2004; Lhuissier et al. 2012; Richalet and Lhuissier 2015; Richalet et al. 2020). Beyond HVR, other physiological responses at high altitude may be impacted by hormone levels. For example, estrogen could enhance capillary permeability and increase plasma volume (Tollan et al. 1992). As already discussed (Horakova et al. 2024), this mechanism may induce fluid retention, which have been associated with acute mountain sickness (AMS) (Hackett et al. 1982). Alternatively, the estrogen’s vasodilatory effects (Behan and Wenninger 2008) may mitigate hypoxic pulmonary vasoconstriction (Lahm et al. 2007), potentially lowering the incidence of AMS and high altitude pulmonary edema in eumenorrheic women (EW). Estrogen has also been shown to increase cerebral blood flow (Krause et al. 2006) potentially affecting AMS occurrence. Aging-induced brain size reduction might contribute to increased tolerance to altitude-induced brain swelling, leading to decreased intracranial pressure (Ross 1985; Roach and Hackett 2001) and ultimately decreased AMS incidence in PW. Overall, despite these differences between EW and PW, AMS incidence is generally reported to be similar between the two groups (Richalet et al. 2020; Gardner et al. 2024) and age has been mostly negatively or not correlated with AMS incidence (Wu et al. 2018; Gianfredi et al. 2020). However, in most previous studies, EW menstrual cycle phases were either not reported or not determined with gold-standard methods (i.e., with ovarian hormones measurements). Moreover, the measurements of anxiety level (Bian et al. 2016; Oliver et al. 2012), inflammatory status (Boos et al. 2016) and redox balance regulation (Bailey et al. 2009) are potentially influencing the incidence and/or severity of AMS and may also differ between age groups.

To our knowledge, the effectiveness of hypoxic tolerance assessment by simultaneous measurement of ventilation (V̇E), peripheral oxygen saturation (SpO_2_), and cerebral oxygenation at rest or during exercise in normobaric hypoxia (NH) has never been directly compared between EW and PW. Therefore, the present study investigated (i) the physiological characteristics and responses at rest and during exercise in NH between EW in the mid-luteal phase and PW; and (ii) whether AMS incidence when exposed for one night at high altitude is influenced by menopause. These two hypotheses have never been tested concomitantly. Based on the assumption that elevated hormonal levels may stimulate the response to hypoxia, we hypothesized that EW in the mid-luteal phase may exhibit higher HVR and lower AMS incidence in hypobaric hypoxia.

Methods

### Participants

Thirteen EW and fifteen PW took part in this study. The characteristics of the EW have been already presented in a previous study (Citherlet et al. 2024). The initial call was in the local newspaper and more than 230 women volunteered. Final selection was based on the availability of the participants and on the following inclusion criteria: be healthy, non-smoker, with a body mass index < 30 kg/m^2^, not pregnant, with no recent (less than one month) extended stay in altitude (> 2500 m), without known history of low iron status, not a competitive swimmer or breath-hold diver and free of cardiovascular, respiratory (including asthma), or central nervous system disease or β-blocker medication use. EW had also to be between 20 and 40 years old and naturally menstruating (with menstrual cycle lengths ≥ 21 days and ≤ 35 days, to not have self-reported or diagnosed menstrual irregularities such as amenorrhea, anovulation, oligomenorrhea, etc., and to not use hormonal contraceptives or hormone replacement therapy three months before recruitment). PW had to be between 60 and 70 years old, without surgically-induced menopause and to have experienced 12 consecutive months of amenorrhea.

### Study design

**Fig. 1:**
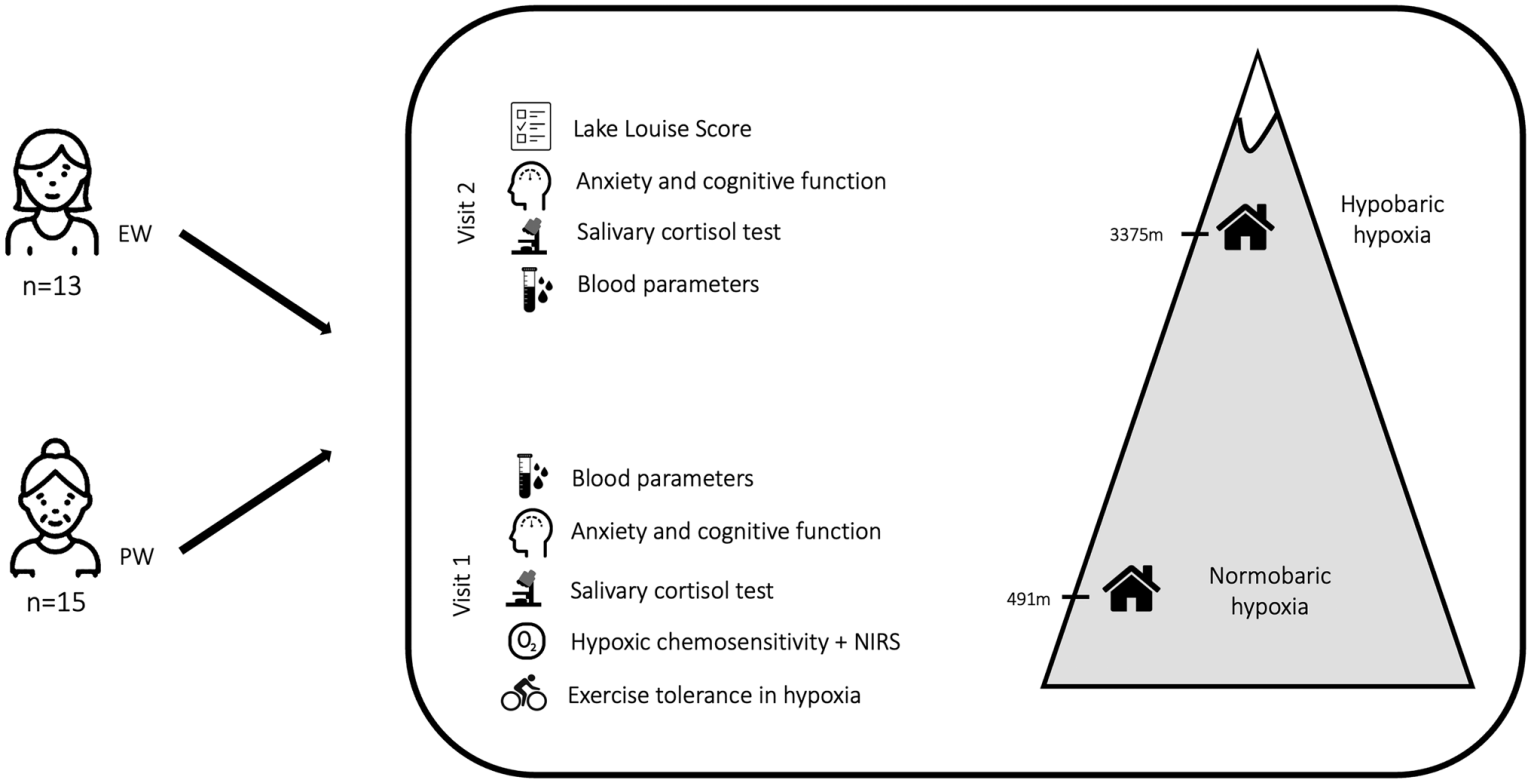
Study design. *EW* eumenorrheic women, *PW* postmenopausal women. *NIRS* Near infrared spectroscopy

The study involved two separate visits (Fig. 1). The first visit was performed in normoxia and in a normobaric hypoxic chamber (LowOxygen Systems, Berlin, Germany) located at Sion, Switzerland (terrestrial altitude: 485 m). EW were tested during the mid-luteal phase, which exhibits the highest levels of estradiol and progesterone. Therefore, the visits were planned to occur between 68 and 82% of the predicted cycle length, calculated using the calendar-based method starting from the first day of their previous menstruation. Women had an average cycle length of 27 ± 3 days and were tested after 76 ± 9% of their cycle length.

The second visit involved an overnight stay in hypobaric hypoxia at Torino Hut in Italy (3375 m).

Although the potential differences between normobaric and hypobaric hypoxia remain debated (Millet and Debevec 2020; Richalet 2020), the prediction of AMS at high-altitude is commonly performed with chemosensitivity tests using normobaric hypoxia (i.e., lower inspired oxygen fraction). Moreover, without access to a hypobaric chamber some tests performed during visit one would not be possible in hypobaric hypoxia for logistical reasons.

The study protocol was approved by the Canton Vaud Research Ethics Committee (2022-00178), adhered to the Declaration of Helsinki's guidelines, and all participants gave written informed consent before being included.

#### Visit 1: normoxia and normobaric hypoxia

During the first visit, participants underwent a series of tests conducted in 2 h sessions in normoxia followed by exposure to NH. These sessions were always administered by the same investigator and followed the predetermined order outlined below.

##### Blood sampling

A total of 6 mL of venous blood was collected from the antecubital vein. The sample was promptly centrifuged for 7 min and 30 s at a speed of 3000 G (Sorvall ST8, Thermo Scientific, Tewksbury, MA, USA). The serum was then transferred into microtubes and frozen at − 80 °C to ensure optimal preservation.

The levels of serum estradiol, serum progesterone (McNulty et al. 2020), C-reactive protein (CRP), and oxidative stress were assessed. Markers of iron stores, including ferritin, ferrous ion were also monitored to interpret the potential confounding effects on participants' physiological systems (Ryan et al. 2021).

The concentrations of estradiol and progesterone were determined using a competitive enzyme-linked immunosorbent kit (Estradiol ELISA kit, MyBioSource^®^, San Diego, CA, USA; Progesterone ELISA kit, Abnova^®^, Taipei City, Taiwan).

For CRP concentrations, a sandwich enzyme-linked immunosorbent kit (CRP ELISA kit, Abnova^®^, Taipei City, Taiwan) was employed. Ferritin concentrations were determined using a sandwich enzyme-linked immunosorbent kit (Ferritin ELISA kit, Elabscience^®^, Houston, Texas, USA), and ferrous iron concentrations were measured using a colorimetric assay kit (Ferrous iron colorimetric assay kit, Elabscience^®^, Houston, Texas, USA).

The oxidative stress markers, namely advanced oxidation protein products (AOPP), catalase, glutathione peroxidase (GPx), myeloperoxidase, nitrites, nitrates, total nitrite and nitrate, total superoxide dismutase, and xanthine oxidase were measured as previously indicated by Manferdelli et al. (2023) while ferric reducing antioxidant power and malondialdehyde (MDA) were measured as previously reported by Martin et al. (2020).

##### Anxiety

Anxiety levels were measured using the State-Trait-Anxiety-Inventory (STAI; Spielberger 1983). This inventory consists of 20 items that assess the individual's current anxiety state, referred to as “State Anxiety” (S-Anxiety), as well as 20 items that evaluate background sustained anxiety, referred to as "Trait Anxiety" (T-Anxiety). The total score for each questionnaire (S-Anxiety + T-Anxiety), ranges from 20 to 80, with higher scores indicating higher levels of anxiety.

##### Hypoxic ventilatory response

Hypoxic ventilatory response was evaluated using the pure nitrogen breathing test (N_2_T) (Solaiman et al. 2014). After 3 min of baseline in a semi-recumbent position, participants were exposed to a series of 10 periods of 100% nitrogen inhalation (1–8 consecutive breaths) in a randomized order and interposed by ≥ 2 min of ambient air. Transitions between room air and nitrogen breathing were accomplished using a 3-way valve, which was connected to the participant via a gas mask and out of sight. HVR was calculated by plotting the lowest finger SpO_2_ (WristOx^®^ 3150 Nonin, Medical Inc., USA) against the highest V̇E (Quark metabolic cart, Cosmed, Rome, Italy) for each nitrogen exposure and taking the absolute value of the slope of the V̇E-SpO_2_ relation.

##### Cerebral oxygenation

Prefrontal cerebral oxygenation was concomitantly monitored by near-infrared spectroscopy (NIRS). The tissue saturation index (TSI), and relative changes in deoxygenated hemoglobin and oxygenated hemoglobin concentrations were recorded using a NIRS device (PortaLite, Artinis Medical Systems, Elst, The Netherlands). The device consisted of 3 dual-wavelength (760 and 850 nm) light transmitters-channels distanced by 30, 35, and 40 mm from a receiving optode, positioned on the prefrontal cortex, and surrounded by a bandage to ensure optimal contact and minimize light interference.

##### Responses to acute normobaric hypoxia

Physiological responses at rest and during exercise were evaluated using a cycling exercise which consisted of 5 min of rest (cycling position) followed by 5 min of cycling at 1.5 W/kg in hypoxia (F_i_O_2_ = 14%, simulated altitude of 3500 m). During exercise, measurements of earlobe SpO_2_ and heart rate (HR) were obtained using a pulse oximeter (WristOx 3150, Nonin Medical, Plymouth, MN, USA). V̇E was measured using a portable gas analyzer (MetaMax 3B, Cortex Biophysik GmbH, Leipzig, Germany).

SpO_2_ and HR values were averaged over the last 10 s of both the rest and exercise periods, while V̇E was averaged over the last 10 breaths. Cycling efficiency was calculated as previously described elsewhere (Moseley and Jeukendrup 2001).

#### Visit 2: high altitude

In the second visit, all participants had their cortisol (as described below) and hormonal levels (as described for visit 1) assessed at low altitude (485 m) prior to the ascent. Subsequently, they traveled around 4 pm to Torino hut by cable car (ascent duration: 15–20 min) and spent one night at this altitude. Before bedtime (6 h after arrival), AMS was assessed using the 2018 Lake Louise Scale and AMS was identified at the threshold of three points, including headache (Roach et al. 2018), while SpO_2_ and HR were measured using a finger pulse oximeter (WristOx 3150, Nonin Medical, Plymouth, MN, USA). The following morning (18 h after arrival), AMS, SpO_2_, and HR were assessed again along with anxiety (as described in visit 1) and cortisol levels.

Cortisol levels were evaluated through saliva samples collected at 0 min (C1), 30 min (C2), and 45 min (C3) after awakening. Participants were instructed to rinse their mouths with water and then move a cotton swab around the mouth to collect saliva for 2 min without chewing. No liquid intake (except water), food consumption, or smoking was authorized during sampling. The collected samples were stored at – 20 °C, transferred to – 80 °C for long-term storage, and transported to the laboratory for analysis. During transportation, the samples were maintained on ice to ensure their integrity. Cortisol concentrations were measured using a competitive enzyme-linked immunosorbent kit (Salivary cortisol ELISA kit, ELISA kit, Abnova^®^, Taipei City, Taiwan). Two indices were calculated: First awakening sample (C1) and the area under the cortisol rise curve relative to the ground (AUC-G; [(C1 + C2)/2]·30 + [(C2 + C3)/2]·15).

### Statistical analysis

A statistical power analysis was conducted using G*Power 3.1 to determine the appropriate sample size for this study. A-priori sample size was derived from HVR data in Takano (1984), which reported a significant increase in hypoxic sensitivity from the follicular phase to the luteal phase of the menstrual cycle in 10 women. Using a significance level of 0.05 and a desired power of 0.80, the power analysis indicated that a sample size of nine participants (Cohen’s d effect size of 1.17) would be necessary to detect statistically significant differences in HVR between the two phases. To our knowledge, there are no data available for such calculation of the required sample sizes by directly comparing HVR between EW and PW. Therefore, although the variations in hormonal levels across the menstrual cycle are likely lower than the variations occurring with menopause, we increased the sample size by 50%, corresponding to 13 in each group.

A t-test for independent samples was conducted to compare EW and PW characteristics, pure nitrogen breathing test parameters and oxidative stress responses. Levene’s test for equality of variances was employed to determine if the assumption of equal variances was violated and, in that case, Welch’s t-test was used. Normality was checked using the Shapiro–Wilk test with no further analysis required.

A two-way repeated measures Anova for ventilation, HR, saturation, AMS score was applied. When the assumption of sphericity was violated, the Greenhouse–Geisser correction was applied. When a significant Anova result was found, Bonferroni post-hoc test were conducted to identify specific differences.

All statistical analyses were performed using SPSS, version 26 (IBM Corp., Armonk, NY, USA). The significance level for all tests was set at P < 0.05. Data are shown as mean ± standard-deviation (SD) in manuscript and tables and as mean ± standard error of the mean (SEM) in figures.

## Results

### Participants

The participants’ characteristics are shown in Table 1.

**Table 1:** Participants’ characteristics

	Eumenorrheic women	Postmenopausal women
Age (year)	32 ± 8	63 ± 2*
Weight (kg)	63 ± 10	56 ± 8
Height (cm)	167 ± 8	163 ± 5
Ferritin (ng/mL)	35 ± 25	68 ± 37*
Ferrous iron (μmol/L)	19 ± 18	18 ± 19
C-reactive proteins (mg/L)	1.7 ± 1.5	1.3 ± 1.2

Mean ± SD

*indicates differences between eumenorrheic women and postmenopausal women (p < 0.05)

**Fig. 2:**
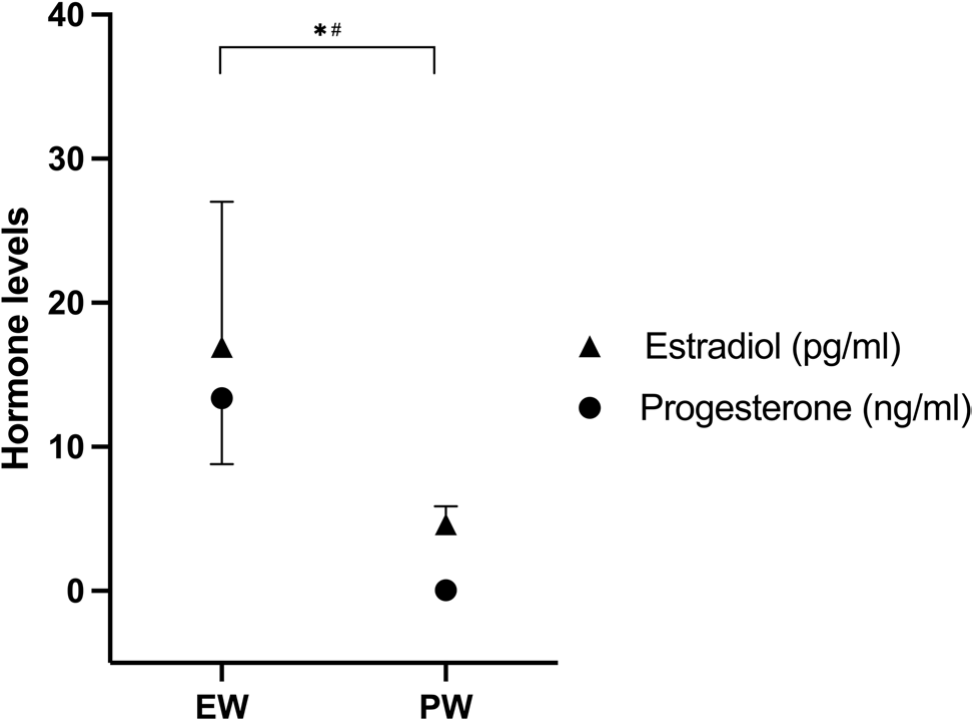
Ovarian hormones in eumenorrheic women (EW) during their mid-luteal phase and in postmenopausal women (PW). Each symbol represents mean ± 95% confidence intervals (too small to be seen in PW progesterone). * indicates a difference for estradiol and # indicates a difference for progesterone

Estradiol and progesterone levels were significantly higher in EW than in PW (Fig. 2).

During the N_2_T, PW had lower V̇E at baseline (P = 0.027), a lower range of V̇E (P = 0.016), and a lower range of SpO_2_ (P < 0.001) but no significant difference in HVR or any other parameters of the test (Table 2). No significant differences in V̇E, SpO_2_ and HR both at rest or during exercise were observed between the two groups (Fig. 4). Cycling efficiency (0.20 ± 0.02 vs. 0.19 ± 0.03%) and power output (82 ± 16 vs. 84 ± 9 W) in acute normobaric hypoxia were not significant different between EW and PW.

**Table 2:** Pure nitrogen breathing test parameters

	*Eumenorrheic women*	*Postmenopausal women*
Baseline SpO_2_ (%)	95.4 ± 1.5	94.9 ± 1.6
Baseline V̇E (L/min)	**10.0 ± 1.5**	8.5 ± 0.9*
Hypoxic ventilatory response (L/min/%)	– 0.34 ± 0.13	– 0.27 ± 0.15
Difference (min–max) in V̇E (L/min)	**8.2 ± 3.1**	5.4 ± 2.2*
Min SpO_2_ (%)	77.2 ± 6.1	80.2 ± 7.1
Difference (max–min) in SpO_2_ (%)	**18.2 ± 5.3**	14.7 ± 6.4*
Cerebral tissue saturation index slope (%/s)	– 0.19 ± 0.16	– 0.17 ± 0.14
Cerebral tissue saturation index amplitude (%)	– 6.2 ± 3.2	– 6.7 ± 3.9
Cerebral deoxyhemoglobin slope (μM/s)	0.11 ± 0.04	0.08 ± 0.05
Cerebral deoxyhemoglobin amplitude (μM)	4.1 ± 1.3	3.1 ± 1.6
Cerebral oxyhemoglobin slope (μM/s)	– 0.08 ± 0.03	– 0.06 ± 0.04
Cerebral oxyhemoglobin amplitude (μM)	– 2.3 ± 0.9	– 2.2 ± 1.2

Mean ± SD

*SpO*_*2*_ oxygen saturation, *V̇E* minute ventilation

* with values highlighted in bold for differences between eumenorrheic women and postmenopausal women (p < 0.05)

**Fig. 3:**
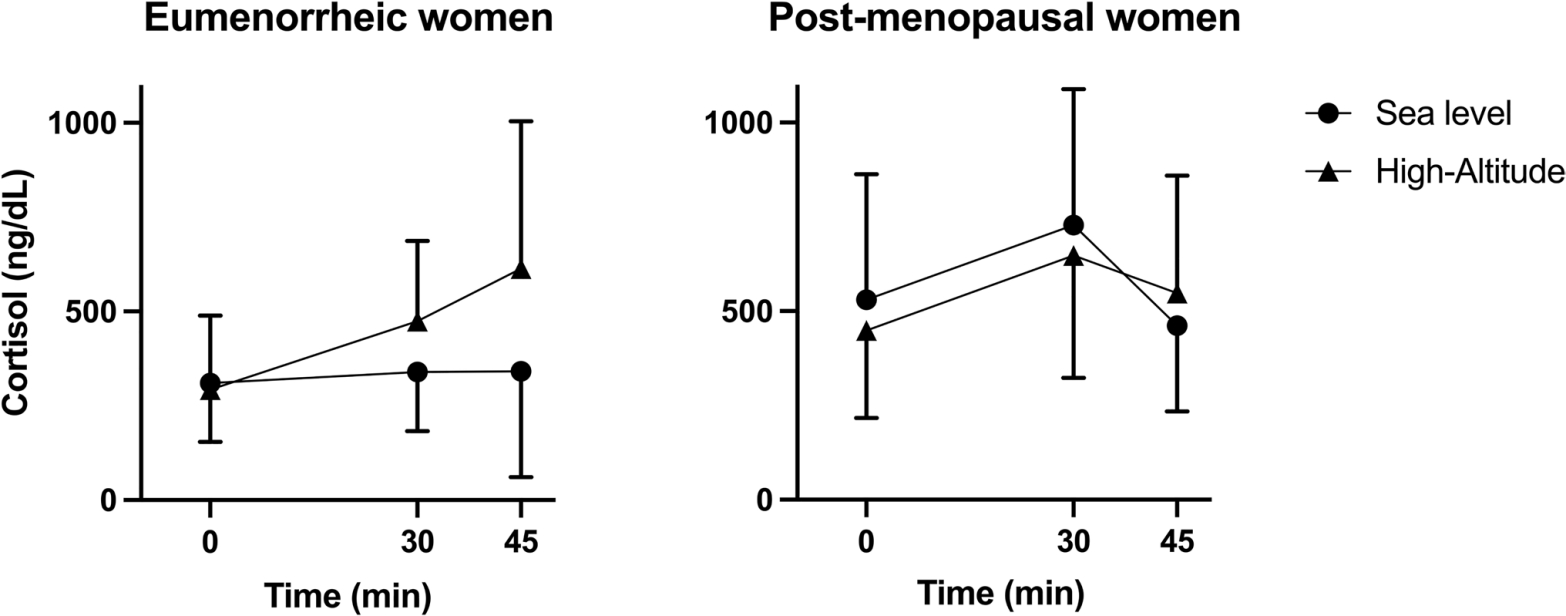
Cortisol levels at low and high altitudes in eumenorrheic women and postmenopausal women. Each symbol represents mean ± 95% confidence intervals

Similar oxidative stress markers (Table 3), cortisol levels (Fig. 3 and Table 4), and anxiety scores (Table 4) were found between EW and PW.

**Table 3:** Oxidative stress parameters

	Eumenorrheic women	Postmenopausal women
Advanced oxidation protein products (μmol/L)	259 ± 64	275 ± 62
Catalase (μmol/L/min)	9.4 ± 5.1	7.8 ± 3.2
Ferric reducing antioxidant power (μmol/L)	1256 ± 244	1187 ± 253
Glutathione peroxidase (μmol/L/min)	4.8 ± 0.7	4.5 ± 0.9
Malondialdehyde (μmol/L)	2.3 ± 1.1	2.4 ± 0.7
Myeloperoxidase (μmol/L/min)	105 ± 112	68 ± 35
Nitrites (μmol/mL)	17 ± 12	15 ± 14
Nitrates (μmol/mL)	45 ± 19	38 ± 16
Total nitrite and nitrate (μmol/mL)	28 ± 11	23 ± 9
Total superoxide dismutase (μmol/L/min)	17 ± 10	11 ± 7
Xanthine oxidase (μmol/L/min)	8 ± 2	8 ± 3

Mean ± SD

**Table 4:** Cortisol and anxiety

	Eumenorrheic women	Postmenopausal women
Cortisol C1 at low altitude (ng/dL)	310 ± 258	531 ± 600
Cortisol AUCG at low altitude (ng/dL.min)	330 ± 218	618 ± 463
Cortisol C1 at high altitude (ng/dL)	293 ± 309	449 ± 420
Cortisol AUCG at high altitude (ng/dL.min)	446 ± 344	565 ± 396
S-Anxiety at low altitude	32.8 ± 6.5	28.9 ± 6.6
T-Anxiety at low altitude	38.0 ± 8.5	34.1 ± 7.8
S-Anxiety at high altitude	28.5 ± 5.5	28.3 ± 7.3
T-Anxiety at high altitude	35.5 ± 8.3	35.1 ± 8

Mean ± SD

*C1* first cortisol sample, *AUG-C* cortisol awakening response, *S-Anxiety* state anxiety inventory, *T-Anxiety* Trait anxiety inventory

**Fig. 4:**
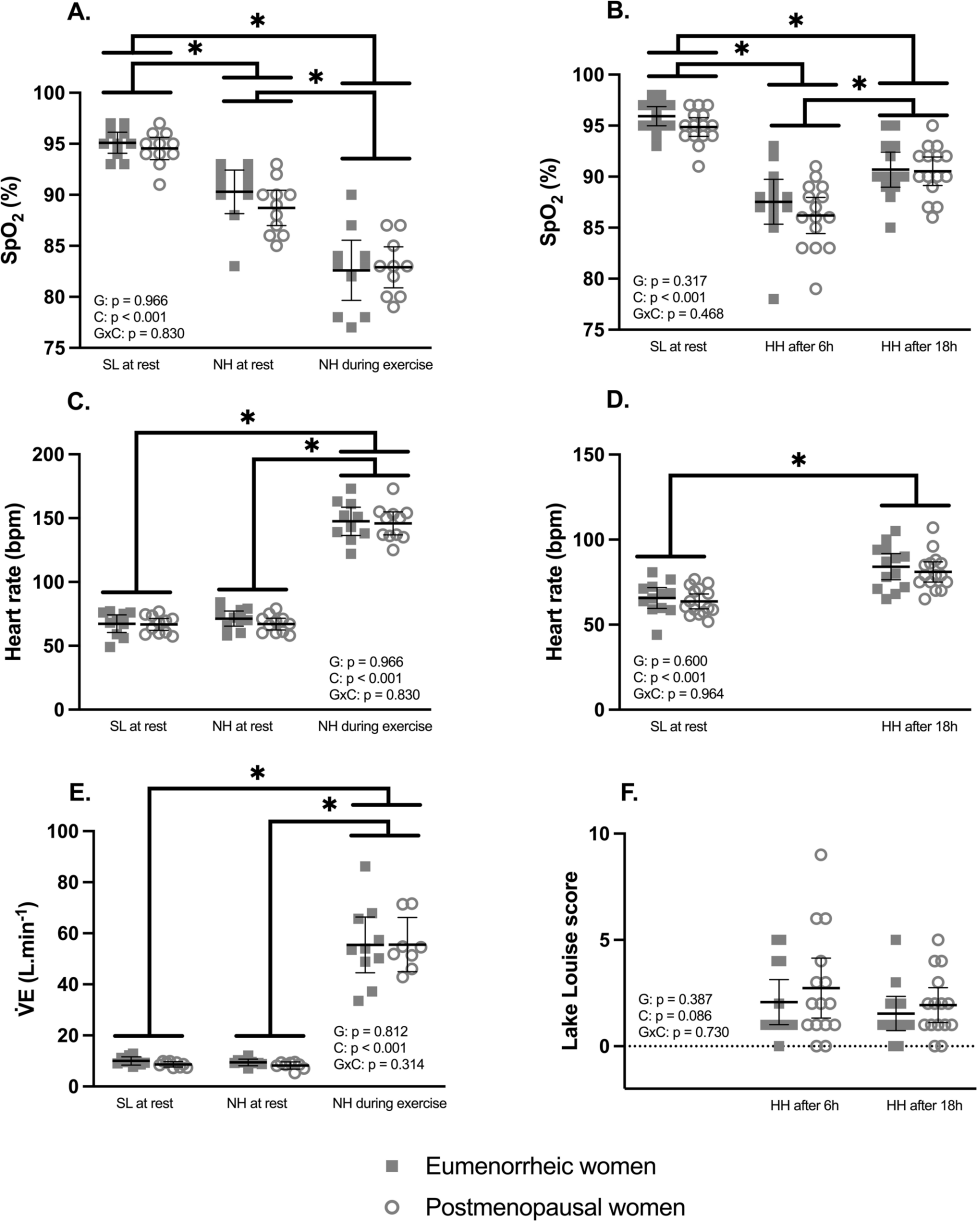
Responses to acute normobaric hypoxia (NH, panel **A**, **C**, and **E**) and response to prolonged hypobaric hypoxia (HH, panel **B**, **D**, **F**) in eumenorrheic women during their mid-luteal phase and in postmenopausal women. SL (sea-level), SpO_2_ (peripheral oxygen saturation), V̇E (ventilation), G (ANOVA group main effect), C (ANOVA condition main effect), GxC (ANOVA group x condition interaction effect). Mean ± 95% confidence intervals. * for differences between conditions when eumenorrheic and postmenopausal women are pooled (p < 0.05)

AMS scores (Fig. 4F) and AMS incidence were not statistically different between EW and PW, with 4 out of 13 (31%) in EW and 6 out of 15 (40%) in PW experiencing AMS after 6 h (P = 0.611), and 2 out of 13 (15%) in EW and 4 out of 15 (27%) in PW after one night (P = 0.468) at high altitude (Fig. 4). In PW, the AMS + group at + 6 h exhibited a lower HR at rest in normoxia (58.2 ± 4.2 vs 67.4 ± 7.8; P = 0.020) and in NH (59.2 ± 0.9 vs 69.9 ± 5.5; P = 0.004), a higher T-Anxiety in NH (40.0 ± 6.8 vs 29.6 ± 5.2; P = 0.007), a lower GPx (3.9 ± 0.7 vs 4.9 ± 0.8; P = 0.046) and a lower MDA (1.8 ± 0.2 vs 2.8 ± 0.6; P = 0.005). No significant differences were observed between AMS + and AMS− groups in EW.

## Discussion

This study aimed to compare the biological differences and the physiological responses at rest and during exercise in NH between EW and PW and to evaluate the AMS incidence in these two subgroups when exposed to high altitude. The main results of the present study are: (i) EW exhibited similar HVR than PW in NH; (ii) measurements of HR/SpO_2_ levels at high altitude were similar between EW and PW; (iii) AMS incidence was similar in women of both age-groups when exposed to high altitude.

(i) Ventilatory responses

EW and PW exhibited no significant difference in HVR, which is corroborated by prior investigations at rest (Pokorski and Marczak 2003; Pokorski et al. 2004). The literature on HVR during exercise is more inconsistent. Richalet et al. (2020) found comparable exercise HVR and SpO_2_,while Puthon et al. (2017) reported similar HVR at low and moderate exercise intensities but higher in EW during maximal exercise. Lhuissier et al. (2012) and Richalet and Lhuissier (2015) reported similar SpO_2_ and HVR between EW and trained PW, but a decreased HVR in untrained PW compared to EW. While it could be expected that decreased hormonal levels would blunt HVR, it has also been suggested (Richalet and Lhuissier 2015) that HVR could be increased in the elderly as they have a greater incidence of obstructive sleep apnea (Edwards et al. 2010), which is known to increase hypoxic chemosensitivity (Foster et al. 2005). Overall, available literature suggests that HVR differences are negligible at rest, while during exercise, results are heterogeneous and potentially influenced by the intensity of exertion and the subjects' training status, with higher intensity and lower training status favouring differences.

(ii) Physiological responses to high altitude and incidence of acute mountain sickness

The incidence of AMS (i.e., approximately one-third after 6 h of exposure) aligns with the expected rates for this altitude (Bärtsch and Swenson 2013), and the absence of differences between EW and PW is consistent with previous reports (Richalet et al. 2020; Gardner et al. 2024) and with a recent meta-analysis reporting no correlation between age and AMS (Wu et al. 2018).

HVR quantifies an essential adaptation mechanism to hypoxia by calculating the ratio between the increase in V̇E and the decrease in SpO_2_ during hypoxic exposure. A high HVR value is considered advantageous at high altitudes as it facilitates efficient oxygen delivery to the tissues (Milledge 1987). Yet, in the present study, this parameter and most of the parameters measured either in NH or during the N_2_T test failed to predict AMS. Previous studies investigating the predictive ability of the HVR in determining susceptibility to AMS have produced conflicting outcomes. Some studies have reported successful associations (Hackett et al. 1988; Hu et al. 1982; Rathat et al. 1992; Richalet et al. 1988), but others did not (Milledge et al. 1988, 1991; Bärtsch et al. 2002). The differences have been suggested to be due to the method used to evaluate HVR (West et al. 2012) as in most of these studies the hypoxic stimulus is only a few minutes while longer stimuli (i.e. 20–30 min), as tested by Burtscher et al. (2004), achieved an 86% accuracy in correctly predicting AMS. One cannot rule out that some of these differences might also be related to the use of isocapnic vs poikilocapnic hypoxic ventilatory response testing. Another effective approach demonstrated by Richalet et al. (2021) is combining the HVR with a scoring system (SHAI score) and using an HVR at exercise. HVR is the main physiological risk factor of SHAI (Richalet et al. 2012). The positive predictive of SHAI is 29%, a value reflecting the effectiveness of preventive measures advised to those identified at high risk, who in turn may exercise greater caution. The negative predictive value is notably higher at 81%, indicating a reliable exclusion of low-risk subjects from unnecessary acetazolamide use, underscoring the procedure's efficiency (Richalet et al. 2021).

Beyond HVR, it has been proposed that elements such as anxiety and cortisol could be linked to the incidence of AMS. In the present study and in line with Boos et al. (2018) and Missoum et al. (1992), T-Anxiety at low altitude but not S-Anxiety was higher in AMS + PW, indicating a potential link between anxiety and susceptibility to AMS. The predictive ability of T-Anxiety as opposed to S-Anxiety could be attributed to its nature, which represents more stable individual anxiety levels over time.

The cortisol levels did not predict AMS which is in line with Woods et al. (2012) but not with other works that found increased cortisol levels in AMS + participants (Richalet et al. 1989; Estoppey et al. 2019; Gatterer et al. 2019). Different results can be due to exposure duration, altitude level, and levels of physical exertion.

### Limitations

It is worth noting that this study had some limitations. This study is underpowered to compare AMS incidence between EW and PW. Overall, the present study has to be considered as a negative study showing no significant difference in ventilatory and cerebral blood flow responses between pre- and post-menopausal women.

The N_2_T ability the predict AMS is likely limited due to the influence of random V̇E variations during short nitrogen exposures, and because individual differences in breathing frequency lead to differences in SpO_2_ decreases (< 50% for some individuals with very low breathing frequencies). Moreover, HVR has been shown to be biphasic (i.e., with an acute HVR increase followed by a hypoxic ventilatory decline) (Ainslie et al. 2013), and AMS typically develops over hours and/or higher physical activity levels. Finally, this study assesses the impact of decreased hormonal levels at menopause, but the effects evaluated are intrinsically also induced by age, which is a limit of the model of menopause to study hormonal changes. Beyond the influence of menopause on the hormonal status of women, aging induces several mechanisms (e.g., lung elasticity, respiratory muscle fatigability, diffusion capacity…) that potentially modify the ventilatory responses.

## Conclusion

This study demonstrates that menopause, despite lowering estradiol and progesterone levels, does not affect physiological responses to hypoxia (e.g., HVR) or AMS incidence. Overall, the present results showed no significant difference in ventilatory and cerebral blood flow responses between EW and PW, suggesting that aging women should not be more concerned than their younger counterparts about getting exposed to altitude.

## Data Availability

Data are available from the corresponding author upon reasonable request.
